# Pharmacokinetically guided algorithm of 5-fluorouracil dosing, a reliable strategy of precision chemotherapy for solid tumors: a meta-analysis

**DOI:** 10.1038/srep25913

**Published:** 2016-05-27

**Authors:** Luo Fang, Wenxiu Xin, Haiying Ding, Yiwen Zhang, Like Zhong, Hong Luo, Jingjing Li, Yunshan Yang, Ping Huang

**Affiliations:** 1 Laboratory of Clinical Pharmacy, Zhejiang Cancer Hospital, Hangzhou, China; 2 Department of Chemotherapy, Zhejiang Cancer Hospital, Hangzhou, China

## Abstract

Precision medicine characterizes a new era of cancer care and provides each patient with the right drug at the right dose and time. However, the practice of precision dosing is hampered by a lack of smart dosing algorithms. A pharmacokinetically guided (PKG) dosing algorithm is considered to be the leading strategy for precision chemotherapy, although the effects of PKG dosing are not completely confirmed. Hence, we conducted a meta-analysis to evaluate the effects of the PKG algorithm of 5-fluorouracil (5-FU) dosing on patients with solid tumors. A comprehensive retrieval was performed to identify all of the prospective controlled studies that compared the body surface area (BSA)-based algorithm with the PKG algorithm of 5-FU in patients with solid tumors. Overall, four studies with 504 patients were included. The PKG algorithm significantly improved the objective response rate of 5-FU-based chemotherapy compared with the BSA-based algorithm. Furthermore, PKG dosing markedly decreased the risk of total grade 3/4 adverse drug reactions, especially those related to hematological toxicity. Overall, the PKG algorithm may serve as a reliable strategy for individualized dosing of 5-FU.

Currently, cancer therapy is progressing toward a new era of precision medicine with the implementation of novel biomarkers to identify patients, dose with the right drugs, and predict an individual’s response^1^,^2^,^3^. Precision medicine should focus on “precision”, i.e., administration of a precise dose, in contrast with “stratified” medicine, which focuses on prescribing the right drug for patients^1^,^3^. However, precision dosing is hampered by a lack of smart algorithms.

Within the past twenty years, a smart strategy for individual dosing based on pharmacokinetic (PK) profiling of antineoplastic agents has been developed because systemic exposure is a direct biomarker of the clinical response^4^. Moreover, pharmacokinetically guided (PKG) algorithms are increasingly appreciated as an effective method to personalize the dose of both chemotherapy and targeted therapy^5^,^6^,^7^. The drug 5-fluorouracil (5-FU) is considered to be a vanguard drug of pharmacokinetically guided chemotherapy. The dosing of 5-FU is usually based on patients’ body surface area (BSA). However, there is no strong correlation between the plasma clearance of 5-FU and BSA^8^,^9^. The BSA-based dosing algorithm leads to both great variability of individual systemic exposure (up to 100-fold in systemic clearance) and inappropriate dosage (40%–60% of patients with an underdose and 10%–20% of patients with an overdose)^10^. As a result, patients show variations in the pharmacodynamics of 5-FU. This variability of systemic exposure can be attributed to various factors, such as age, gender, disease state, organ function and enzymes involved in 5-FU metabolism, especially dihydropyrimidine dehydrogenase (DPD)^11^,^12^,^13^. Due to the low weight of a single factor in the variability of individual systemic exposure, it is not feasible to personalize the dose on the basis of only a single factor. Instead, the PKG algorithm of 5-FU based on systemic exposure has been proven to minimize the pharmacokinetic variability of 5-FU, keep individual systemic exposure within the optimal range and contribute to an improvement in efficacy and a decrease in toxicity in several clinical studies^14^,^15^,^16^,^17^,^18^,^19^,^20^,^21^,^22^,^23^,^24^,^25^,^26^.

However, the PKG algorithm has not yet been fully implemented in daily oncology practice, because of several disadvantageous factors. Above all, the lack of high-quality evidence is a primary limitation, because inconsistent results have been reported across studies^14^,^15^,^16^,^17^,^27^. In addition, the sample sizes of previous studies have been small, which may have reduced the strength of the results. To confirm the effect of the PKG algorithm of 5-FU, we conducted a meta-analysis to evaluate the efficacy and toxicities of the PKG dosing algorithm for 5-FU in patients with solid tumors.

## Results

### Literature search, identification, and quality assessment

**Figure 1:**
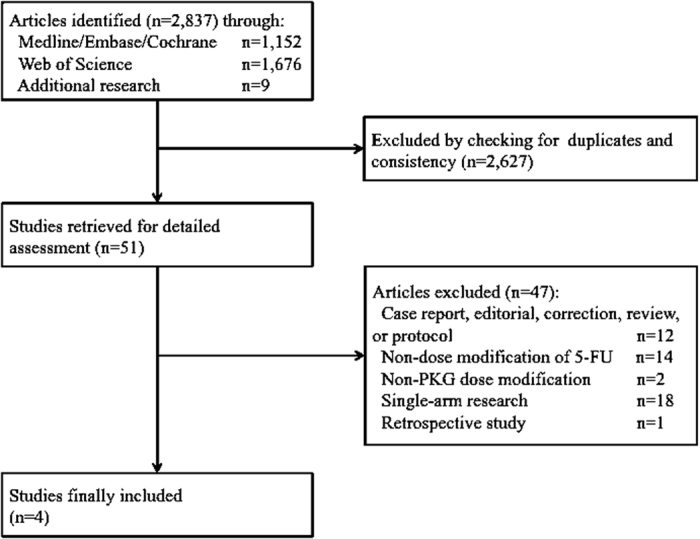
A flow diagram of the procedure for the literature search.

A total of 2,837 studies were initially collected from the literature search, and 51 potentially relevant papers were identified after the abstracts were scrutinized. Among these citations, 47 studies were excluded for the following reasons: not original research (such as case reports, editorials, corrections, reviews, or protocols), not dose-modification studies, not PK-dependent dose adjustment studies, not controlled studies, and not prospective studies. Consequently, four studies were included in the present meta-analysis^14^,^15^,^16^,^27^ and are shown in Fig. 1.

### Study characteristics

The main characteristics of the eligible trials are illustrated in Table 1. Four prospective controlled trials were included; one of these focused on locally advanced head and neck cancer^14^, and the others studied colorectal cancer^15^,^16^,^27^. A total of 504 patients, 213 patients for the BSA-based dosing (BSA arm) and 291 patients for the PKG dosing (PKG arm), were involved in the analysis, and all of the patients were treated with 5-FU-based regimens (FOLFOX^16^, Leucovorin/5-FU^15^,^27^, or cisplatin/5-FU^14^). The dosage of 5-FU was based on the BSA values of patients in the BSA arm and the pharmacokinetic profiles of 5-FU (area under the curve (AUC) or steady-state concentration) in the PKG arm. The clinical response and grade 3/4 adverse drug reactions (ADRs) were reported in all of the studies. However, the studies lacked appropriate descriptions of the randomization procedures, the double-blind methodology, the number of drop-outs (only described by Gamelin^15^), and intent to treat analysis. Thus, the methodological quality of the included studies was low.

### Clinical response

**Figure 2:**
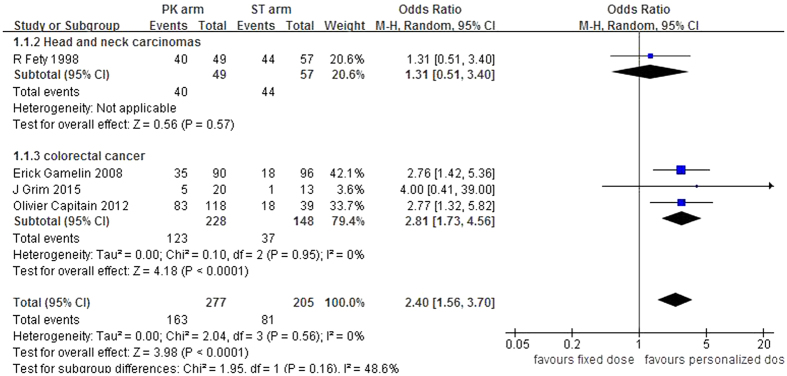
Forest plots of the odds ratio for the overall clinical response.

All of the trials reported the objective response rate (ORR, from 38.9% to 81.6% in the PKG arm and from 18.8% to 77.2% in the BSA arm) or the pathological complete response rate (pCR, 25% in the PKG arm and 7.69% in the BSA arm, as shown in Table 1). The pooled analysis indicated that the PKG strategy significantly improved the overall response rate compared with the BSA arm (odds ratio (OR) = 2.40, 95% CI: 1.56–3.70, *p* < 0.0001). There was no significant inter-study heterogeneity (I^2^ = 0%, *p* = 0.56). An additional subgroup analysis according to the type of cancer was performed, and the pooled result showed that PKG dosing of 5-FU improved the ORR of 5-FU-based chemotherapy for colorectal cancer patients (OR = 2.82, 95% CI: 1.73–4.56, *p* < 0.0001; I^2^ = 0%, *p* = 0.95, Fig. 2).

### Survival

Two of four trials reported survival outcomes^15^,^16^. However, only one trial reported survival data for both the BSA arm and the PKG arm: the one-year overall survival (OS) rate increased from 59.5% to 70.5% by PKG dosing, whereas the median OS improved by 6 months (16 months in the BSA arm and 22 months in the PKG arm, *p* = 0.08)^15^. Therefore, the pooled effect on the survival outcome of PKG dosing was not determined in this study.

### Toxicities

**Figure 3:**
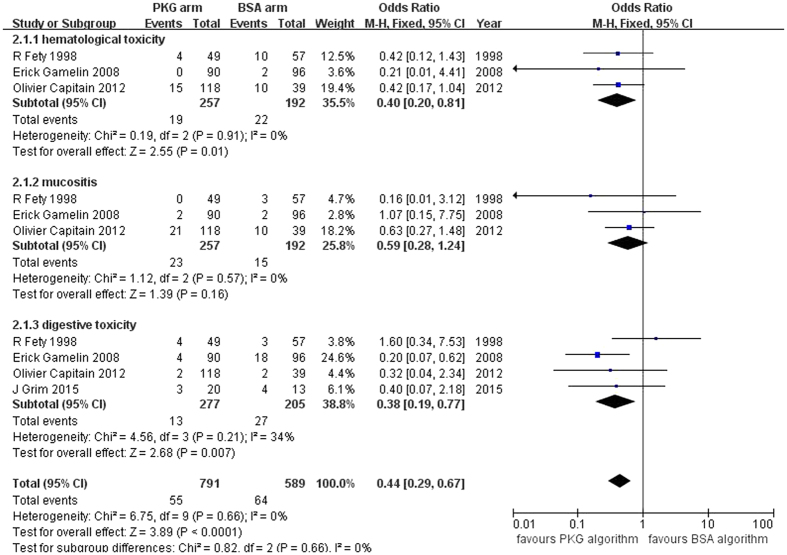
Forest plots of the odds ratio for grade 3/4 ADRs.

All of the trials reported grade 3/4 toxicities of hematological toxicity, mucositis, and digestive toxicity. In addition, the rates of total serious ADRs ranged from 15.4% to 24.7% for the BSA arm and from 6.6% to 20.0% for the PKG arm (Table 1). No significant inter-study heterogeneity was observed (I^2^ = 0%, *p* = 0.66). The fixed-effect pooled estimate showed a decreased risk of grade 3/4 toxicity for the PKG arm compared with the BSA arm (OR = 0.44, 95% CI: 0.29–0.67, *p* < 0.0001, Fig. 3). Furthermore, a subgroup analysis according to the type of ADRs was conducted, and the PKG dosage of 5-FU was found to significantly decrease the risk of grade 3/4 hematological toxicity (OR = 0.40, 95% CI: 0.20–0.81, *p* = 0.01) and digestive toxicity (OR = 0.38, 95% CI: 0.19–0.77, *p* = 0.007). However, the risk of mucositis was not significantly reduced by the PKG algorithm (OR = 0.59, 95% CI: 0.28–1.24, *p* = 0.16). No significant inter-study heterogeneity of the overall pooled or subgroup analysis was observed (I^2^ < 40%)^28^.

### Sensitivity analysis and bias evaluation

The sensitivity analysis was conducted by converting the fixed-effect model to a random-effect model or excluding potentially heterogeneous trials. The results of the meta-analysis by the random-effect model were consistent with those of the fixed-effect model, except for digestive toxicity (I^2^ = 34%, Supplemental Table 1). The impact of the open-label trial on the pooled results was also evaluated^27^. All of the results of the pooled analysis of results excluding the open-label trial were consistent with the primary outcome (Supplemental Table 1). In addition, Egger’s test showed no significant publication bias (*p* = 0.230–0.466).

## Discussion

5-FU has been a leading drug in pharmacokinetically guided chemotherapy because it demonstrates an exposure-response relationship between systemic drug exposure and clinical events; specifically, higher levels of drug exposure result in severe toxic effects, whereas lower levels lead to weak clinical efficacy. Thus, a desired target therapeutic range to balance the positive and negative clinical effects of 5-FU is crucial for the development of PKG algorithms. An impressive body of work has been conducted to evaluate the therapeutic window and to establish an appropriate algorithm of dosing modulation depending on the specific regimen and administration schedule^14^,^15^,^16^,^17^,^18^,^19^,^20^,^21^,^22^,^23^,^24^,^25^,^26^,^27^.

Three sets of PKG algorithms were involved in the included trials. Two algorithms were based on the steady-state concentration (C_ss_) of 5-FU with ranges of 50–100 ng/mL for 7-day continuous infusion combined with cisplatin and 2,500–3,000 ng/mL for 8-hour continuous infusion combined with leucovorin. The aim of these two algorithms was to obtain a comparable AUC (12.2–15.9 mg·h/L^27^ *vs.* 20–24 mg·h/L^15^,^17^). The third algorithm was developed for the 5-FU/cisplatin regimen of patients with head and neck cancer and was based on the AUC_0–48h_ and the patients’ elimination profiles of 5-FU: a target range of 10,400–15,600 ng·h/mL and 5,760–8,640 ng·h/mL was used for patients with a fast- (AUC_0–96h_ < 3 × AUC_0–48h_) and slow-elimination profile (AUC_0–96h_ > 3 × AUC_0–48h_), respectively^14^.

As previously described, the PKG algorithm may compromise the clinical efficacy by elevating the dose^10^. In the included studies, the PKG algorithms significantly improved the clinical response rate of colorectal cancer patients by 49.6%–212%^15^,^16^,^27^, whereas this algorithm was not superior to the BSA algorithm for patients with head and neck cancer (ORR = 77.2% in the BSA arm and 81.7% in the PKG arm)^14^. This result may have been due to the effect of dose modulation between the two types of cancer: more than two-thirds of colorectal cancer patients on the PKG arm underwent a dose increase of at least 10%, and the overall dose was increased by 10.47% to 19.33%^15^,^16^; in contrast, the average final dose for head and neck cancer patients was reduced by more than 30% after modulation of the dose^14^.

In addition to having a better clinical effect, the PKG algorithm also reduced the risk of toxicity by keeping the AUC out of the toxic range. The risks of total grade 3/4 toxicity, hematological toxicity, and digestive toxicity were all reduced by approximately 60% (*p* ≤ 0.01) with PKG dosing compared with BSA-based dosing. Coincidentally, the dose reduction of 5-FU occurred for 18.64% of the patients in Capitain’s trial^16^ and 66.6% of the cycles in Fety’s trial^14^. This finding suggests that PKG dosing reduces the risk of ADRs by efficiently staying within a safe AUC range. In addition, the effect of PKG on the risk of digestive toxicity should be further evaluated owing to the inconsistent results of the present sensitivity analysis.

In addition, we included an open-label trial in our pooled analysis^27^. Due to its non-blind design, small size (n = 33), unexpected combined therapy with radiotherapy, and distinguishing clinical outcome evaluation criterion (pCR), the trial was considered to have a potentially high risk of bias. Thus, the effect of this trial on the outcome of the pooled analysis was evaluated by a sensitivity analysis. However, no obvious difference in the outcomes of the analyses including and excluding this trial was observed.

Our analysis does have some limitations. Most importantly, all of the included trials were not of a high methodological quality. The sequence generation, allocation concealment, masking, and selective outcome reporting bias were not available in detail, and the bias of the trials was not completely clear. Second, only one trial reported an integrated survival outcome, and we were unable to acquire the missing survival data from the authors. Therefore, the survival benefit of the PKG algorithm was not evaluated. Third, several confounding factors, such as the different types of solid tumors and various chemotherapy regimens, still exist.

In conclusion, this meta-analysis indicates that the PKG algorithm of 5-FU significantly improves the clinical response and reduced the risk of grade 3/4 toxicity, compared with the BSA-based algorithm, for locally advanced head and neck cancer and colorectal cancer. Thus, our results provide evidence that the PKG algorithm for 5-FU may provide a reliable strategy for precise chemotherapy.

## Methods

### Literature search

The online databases of MEDLINE, EMBASE, Web of Science, and CENTRAL (Cochrane Central Register for Controlled Trials) were searched for this study. The searches were limited to studies published until July 10^th^, 2015. The following text words or MESH headings were combined as the search strategy: (1) cancer: oncology, cancer, tumor, carcinoma, neoplastic, or neoplasms [MESH Terms]; (2) individual dosing: dose (or dosage) combined with tailor (tail*, compensat*, optimi*, adapt*, adjust*, or modulat*), individual*, or personal*; (3) PKG: pharmacokinetic*, monitor, area under the curve, AUC, or concentration; (4) trial designation: control*, group, or arm; and (5) 5-fluorouracil: 5-FU, or fluorouracil. In addition, trial registers, patents, references cited in relative reviews or selected papers, and conference proceedings such as those from the American Society of Clinical Oncology (ASCO), American Association for Cancer Research (AACR), and European Society for Medical Oncology (ESMO) were also searched.

### Study selection, quality assessment, and data extraction

The titles and abstracts of all of the studies were independently inspected and identified by two review authors (W.X.X. and J.J.L.) to establish whether the studies met the inclusion criteria. For potentially relevant papers, or those with insufficient data in titles or abstracts to make a clear decision, the full papers were obtained and then assessed independently. The third review author (Y.S.Y.) arbitrated any disagreements between the first two review authors. Studies meeting all of the following eligibility criteria were included: (1) prospective controlled trials; (2) treatment with 5-FU-based regimens for solid tumors; (3) comparison of the PKG dose algorithm of 5-FU (including based on drug concentration, area under the curve, and clearance) with a conventional BSA-based algorithm (dose adjustment for intolerable toxicity was acceptable); and (4) clear and reliable clinical outcomes including clinical response and ADRs.

The quality of each included study was assessed independently by two authors (Y.W.Z. and H.Y.D.) in accordance with the Cochrane Handbook for Systematic Reviews of Interventions^28^. This assessment focused on the following six domains: sequence generation, allocation concealment, blinding, completeness of outcome data, risk of selective outcome reporting, and the risk of other potential sources of bias.

Two review authors (L.F. and H.Y.D.) independently extracted information regarding the patients, trials, treatments, and clinical outcomes from each trial. Authors of the trials were contacted for clarification or supplementation of missing information. The two sets of data from the two review authors were pooled and checked for consistency. Any disagreements were discussed and assessed by the third review author (P.H.).

### Data synthesis and analysis

The ORR and risk of grade 3/4 ADRs were pooled and analyzed using Review manager software (Version 5.2, The Cochrane Collaboration, Copenhagen, Denmark). The main results are displayed in the forest plots. A series of predetermined subgroup analyses were conducted according to the types of cancer (colorectal cancer or head and neck cancer) and ADRs (hematological toxicity, mucositis, and digestive toxicity). The inter-study heterogeneity was evaluated with the *v*^*2*^-based Q statistic and quantified by the I^2^ statistic^29^,^30^. If heterogeneity was not considered to be statistically significant (*p* > 0.10 or I^2^ < 40%), the data were analyzed using a fixed-effect model; otherwise, a random-effect model was used.

The presence of publication bias was evaluated by Egger’s test. A series of sensitivity analyses were performed to determine the impact of pooled models or trials with incompatible factors on the overall results^31^. The OR was calculated for all of the analyses. A statistical test with a *p-*value less than 0.05 was considered significant.

## Additional Information

**How to cite this article**: Fang, L. *et al.* Pharmacokinetically guided algorithm of 5-fluorouracil dosing, a reliable strategy of precision chemotherapy for solid tumors: a meta-analysis. *Sci. Rep.* **6**, 25913; doi: 10.1038/srep25913 (2016).

## Supplementary Material

Supplementary Information

## Figures and Tables

**Table 1:** The characteristics of the eligible trials.

Characteristics	Author (year)							
	R Fety (1998)^14^		E Gamelin (2008)^15^		O Capitain (2012)^16^		J Grim (2015)^27^	
	BSA arm	PKG arm	BSA arm	PKG arm	BSA arm	PKG arm	BSA arm	PKG arm
Trial								
Study design	Multicenter, randomized, controlled		Multicenter, randomized, controlled		Prospective, controlled		Prospective, controlled, open-label	
Sample size	106		208		157		33	
Cancer type	Locally advanced head and neck cancer		Metastatic colorectal cancer		Metastatic colorectal cancer		Locally advanced rectal cancer	
Inclusion and exclusion criteria	**Inclusion criteria:** Age: 18–75 years; PS: 0–2; Measurable disease; Life expectancy: >3 months; Adequate bone marrow function; Adequate liver function; Adequate renal function;		**Inclusion criteria:** Measurable lesion; PS: 0–2; Life expectancy: >3 months; adequate hematopoietic function;		**Inclusion criteria:** Age: >18 years; Measurable lesion; Life expectancy: >3 months; Normal bone marrow and organ function;		**Inclusion criteria:** Adequate hematopoietic function; Adequate renal function; Adequate liver function;	
	**Exclusion criteria:** NA		**Exclusion criteria:** Age: >85 years; Abnormal hematopoietic function; Pregnancy or lactation; History of other malignancy; Neurologic or psychiatric disorder, or cardiac disease or myocardial infarction within the previous 12 months, or serious uncontrolled infections		**Exclusion criteria:** NA		**Exclusion criteria:** Uncontrolled arterial hypertension, therapeutic anticoagulation use, pregnancy or lactation, or need for urgent surgery	
Regimen	5-FU, cisplatin		5-FU, leucovorin		FOLFOX		5-FU, leucovorin	
Patient								
n	57	49	104	104	39	118	13	20
Male/female	52/5	48/1	65/39	61/43	24/15	70/48	9/4	18/2
Age (range)	54 (29–72)	55 (36–69)	71.2 (50–85)	71.5 (52–84)	63 (32–80)	65 (35–81)	67.1	64.6
Algorithm and dose								
Initial dose (mg/m^2^)	4000		1500		2500		2800	
Infusion schedule	96-hour continuous		8-hour continuous		46-hour consecutive		7-day consecutive	
PK parameter	AUC		C_ss_		C_ss_		C_ss_	
Target range	5,760–8,640 ng·h/mL for patients with slow elimination of 5-FU or 10,400–15,600 ng·h/mL for patients with fast elimination of 5-FU		2,500–3,000 ng/mL		2,500–3,000 ng/mL		50–100 ng/mL	
Final relative dose to the initial (%)	91.6	68.9	100	51–220	75–100	60–140	NA	NA
Clinical events, n (%)								
ORR	44 (77)	40 (82)	18 (19)	35 (39)	18 (46)	83 (70)	1 (8)	5 (25)
Total grade 3/4 ADRs	16 (28)	8 (16)	31 (30)	18 (17)	24 (62)	38 (32)	NA	NA
Hematological toxicity	10 (17)	4 (8)	2 (2)	0 (0)	14 (35)	35 (30)	NA	NA
Mucositis	3 (5)	0 (0)	2 (2)	2 (2)	6 (15)	1 (1)	NA	NA
Digestive toxicity	3 (5)	4 (8)	19 (18)	4 (4)	4 (12)	2 (2)	4 (32)	3 (15)
Hand-foot syndrome	NA	NA	7 (7)	11 (11)	NA	NA	NA	NA
Cardiotoxicity	NA	NA	1 (1)	1 (1)	NA	NA	NA	NA

AUC = area under the curve; ADR = adverse drug reaction; BSA = body surface area; C_ss_ = steady-state concentration; FOLFOX = Oxaliplatin, 5-FU, leucovorin; NA = not available; PK = pharmacokinetic; PKG = pharmacokinetically guided; PS = performance status; ORR = objective response rate.
