# Supplementary material for: Pharmacokinetically guided algorithm of 5-fluorouracil dosing, a reliable strategy of precision chemotherapy for solid tumors: a meta-analysis
Source: Sci Rep. 2016 May 27;6:25913. doi: 10.1038/srep25913 (PMC4882511; doi:10.1038/srep25913)
Supplement: Supplementary Information [file srep25913-s1.doc]

Pharmacokinetically guided algorithm of 5-fluorouracil dosing, a reliable strategy of precision chemotherapy for solid tumors: a meta-analysis

Luo Fang ^1^, Wenxiu Xin ^1^, Haiying Ding ^1^, Yiwen Zhang ^1^, Like Zhong ^1^, Hong Luo ^1^, Jingjing Li ^2^, Yunshan Yang ^2^, Ping Huang ^1, *^

^1^ Laboratory of Clinical Pharmacy, Zhejiang Cancer Hospital, Hangzhou, China

^2^ Department of Chemotherapy, Zhejiang Cancer Hospital, Hangzhou, China

^*^ Correspondence and requests for materials should be addressed to P.H. (email: huangping1841@zjcc.org.cn)

Supplemental

Table 1. The results of the sensitivity analysis

| Outcomes | Primary analysis^*^ | |  | Random-effect model | |  |  | Excluding open-label trial | | |
| --- | --- | --- | --- | --- | --- | --- | --- | --- | --- | --- |
|  | OR (95% CI) | *P*-value |  | OR (95% CI) | *P*-value |  |  | OR (95% CI) | *P*-value | |
| Clinical response | | |  |  | |  |  | | |  |
| Overall response | 2.40 (1.56, 3.69) | <0.0001 |  | 2.40 (1.56, 3.70) | <0.0001 |  |  | 2.35 (1.51, 3.64) | <0.0001 | |
| Colorectal cancer | 2.82 (1.74, 4.57) | <0.0001 |  | 2.81 (1.73, 4.56) | <0.0001 |  |  | 2.76 (1.68, 4.53) | <0.0001 | |
| Grade 3/4 toxicity | | |  |  | |  |  | | |  |
| Overall toxicity | 0.44 (0.29, 0.67) | <0.0001 |  | 0.46 (0.30, 0.70) | 0.0003 |  |  | 0.46 (0.30, 0.72) | 0.0006 | |
| Hematological toxicity | 0.40 (0.20, 0.81) | 0.010 |  | 0.41 (0.20, 0.82) | 0.010 |  |  | NA^†^ | NA | |
| Mucositis | 0.59 (0.28, 1.24) | 0.016 |  | 0.62 (0.29, 1.33) | 0.220 |  |  | NA | NA | |
| Digestive toxicity | 0.38 (0.19, 0.77) | 0.007 |  | 0.42 (0.16, 1.09) | 0.080 |  |  | 0.45 (0.12, 1.69) | 0.240 | |

**^*^** Pooled by the fixed-effect model

^†^ Not available
